# COVID-19 in the nervous system: physiopathology and neurological manifestations

**DOI:** 10.1055/s-0043-1769123

**Published:** 2023-07-04

**Authors:** Valder Cavalcante Maia Mendonça Filho, Amanda Gomes de Oliveira, Isabelle de Fátima Vieira Camelo Maia, Ananda Carolina Moraes de Falcone, Beatriz Gioppo Betini, Lucas Bruno Rezende, Fernando Henrique Magri Alves

**Affiliations:** 1 Universidade Federal do Ceará, Faculdade de Medicina de Sobral, Sobral CE, Brazil.; 2 Universidade Federal do Ceará, Faculdade de Farmácia, Odontologia e Enfermagem, Fortaleza CE, Brazil.; 3 Universidade de São Paulo, Faculdade de Medicina, Hospital das Clínicas, São Paulo SP, Brazil.; 4 Universidade de São Paulo, Faculdade de Medicina, Hospital das Clínicas, Departamento de Neurologia, Ribeirão Preto SP, Brazil.; 5 Universidade Federal de Minas Gerais, Hospital das Clínicas, Belo Horizonte MG, Brazil.

**Keywords:** COVID-19, Neurological Manifestations, Viral Tropism, Cytokine Release Syndrome, Covid-19, Manifestações Neurológicas, Tropismo Viral, Síndrome da Liberação de Citocina

## Abstract

**Background** Coronavirus disease 2019 (COVID-19) is a viral infection caused by severe acute respiratory syndrome coronavirus 2 (SARS-CoV-2). Although respiratory manifestations have received greater visibility during the pandemic caused by this virus, numerous neurological complaints related to coronavirus 2 infection have been documented in several countries. These records suggest that this pathogen presents neurotropism, and it can cause different neurological conditions of varying intensity.

**Objective** To investigate the ability of coronavirus 2 to invade the central nervous system (CNS) and its neurological clinical outcomes.

**Methods** The present study consists in a comprehensive literature review of the records available in the PubMed, SciELO, and Google Scholar databases. The descriptors *COVID-19* , *brain* and *physiopathology* , associated with the Boolean operator *AND* , were used in the search. Regarding the inclusion and exclusion criteria, we selected the papers published since 2020 with the highest number of citations.

**Results** We selected 41 articles, most of them in English. The main clinical manifestation associated with COVID-19 patients was headache, but cases of anosmia, hyposmia, Guillain-Barré syndrome, and encephalopathies were also described with considerable frequency.

**Conclusion** Coronavirus-2 presents neurotropism, and it can reach the CNS by hematogenous dissemination and by direct infection of the nerve endings. It causes brain injuries through several mechanisms, such as cytokine storm, microglial activation, and an increase in thrombotic factors.

## Resumo

**Antecedentes** A doença do coronavírus 2019 ( *coronavirus disease 2019* , Covid-19, em inglês) é uma infecção viral provocada pelo coronavírus 2 da síndrome respiratória aguda grave ( *severe acute respiratory syndrome coronavirus 2* , SARS-CoV-2, em inglês). Embora as manifestações respiratórias tenham recebido maior visibilidade ao longo da pandemia provocada por esse vírus, inúmeras queixas neurológicas relacionadas à infecção pelo coronavírus 2 foram documentadas em diversos países. Tais registros sugerem que esse patógeno apresenta neurotropismo, e é capaz de provocar quadros neurológicos diversos e de intensidade variáveis.

**Objetivo** Investigar a capacidade de invasão do sistema nervoso central (SNC) pelo coronavírus 2 e seus principais desfechos clínicos neurológicos.

**Métodos** O presente estudo consiste em uma ampla revisão de literatura a partir dos registros das bases de dados PubMed, SciELO e Google Acadêmico. Nesse contexto, os descritores *COVID-19* , *cérebro* e *fisiopatologia* , associados com o operador booleano *AND* , foram utilizados na busca. Quanto aos critérios de inclusão e exclusão, selecionou-se os trabalhos publicados a partir de 2020 com o maior número de citações.

**Resultados** Foram selecionados 41 artigos, a maioria na língua inglesa. A principal manifestação clínica associada a pacientes acometidos pela COVID-19 foi a cefaleia, mas casos de anosmia, hiposmia, síndrome de Guillain-Barré e encefalopatias também foram descritos com frequência considerável.

**Conclusão** O coronavírus 2 apresenta neurotropismo, e é capaz de alcançar o SNC por disseminação hematogênica e por infecção direta das terminações nervosas. Ele provoca injúria cerebral por meio de variados mecanismos, como tempestade de citocinas, ativação da micróglia e aumento dos fatores trombóticos.

## INTRODUCTION

Although coronavirus disease 2019 (COVID-19) is a viral infection that mainly affects the respiratory tract, the first Chinese reports already described that severe acute respiratory syndrome coronavirus 2 (SARS-CoV-2) had an excellent ability to influence the central nervous system (CNS). 1 The SARS-CoV-2 virus shows great similarity (∼ 79.5%) with the SARS-CoV virus, 2 and there is evidence in the literature that neurotropism (affinity for the neurological system) is common to the coronavirus class, as they share structural similarities and infection mechanisms. Hence, the pathophysiology of the neuroinfection already documented for other coronaviruses can also be applied to SARS-CoV-2. 3 4 5

The brain, as it has a vital physiological function, is protected from injuries of the most diverse origins through different mechanisms. The skull is the main defense against physical injury, and it is reinforced to protect the brain mass. Protection against pathogens and harmful chemical agents is mainly performed by the blood-brain barrier (BBB), which is formed by endothelial cells that selectively regulate the passage of substances present in the bloodstream to the CNS, such as antibodies, the complement system, and coagulation factors. 6 To penetrate such a well-protected organ, coronavirus 2 has different ways of invading the CNS, bypassing the BBB. These neuroinfection mechanisms are mainly mediated by angiotensin-converting enzyme 2 (ACE2), a glycoprotein expressed in the epithelium of the airways, vascular endothelium, heart, kidneys, and brain. 7

## HOW DOES CORONAVIRUS-2 INVADE THE BRAIN?

**Figure 1:**
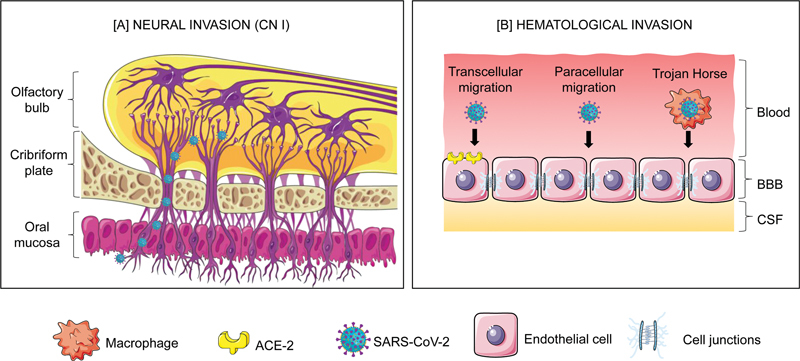
Abbreviations: BBB, blood-brain barrier; CN, cranial nerve; CSF, cerebrospinal fluid. ( **A** ) The virus is inhaled and accesses the CNS through retrograde axonal transport; this mechanism bypasses the BBB. ( **B** ) Different ways that the virus crosses the BBB by the hematogenous route. In both routes, the immune system is activated, triggering an inflammatory picture.

As aforementioned, neurotropism is common in the coronavirus group, and the infection mechanisms include connection, penetration, biosynthesis, maturation, and liberation. 2 Its main cell-invasion mechanism is mediated by ACE2, which is expressed in some brain regions, such as the motor cortex, substantia nigra, olfactory bulb, solitary tract nucleus, and vagus nerve. 7 Coronavirus 2 can enter the CNS in two ways: hematogenous dissemination or direct infection of nerve endings ( Figure 1 , Figure 3 ). Both result in the activation of the host's immune system. 8

### Hematogenous infection

Hematogenous spread occurs when coronavirus 2 migrates from the blood capillaries of the systemic circulation to the brain. However, this organ can regulate the passage of substances present in the bloodstream through the BBB, which is composed of a semipermeable membrane of juxtaposed endothelial cells. Thus, for neuroinvasion via hematogenous dissemination to occur, the virus must first cross this structure. 8 9 10

From this perspective, the access of the pathogen to the CNS can happen through three mechanisms: transcellular migration, paracellular migration, and the “Trojan horse” strategy. In the transcellular migration mechanism, the virus invades the endothelial cells that compose the BBB through the ACE2 glycoprotein. In the paracellular migration mechanism, the virus crosses the juxtaposed junctions between the endothelial cells that compose the BBB. Finally, in the “Trojan horse” strategy, the leukocytes infected by the virus freely cross the BBB. 10 In the CNS, these cells release proinflammatory cytokines, such as tumor necrosis factor-α (TNF-α), which damages oligodendrocytes, and C-C motif chemokine ligand 5 (CCL5), C-X-C motif chemokine ligand 10 (CXCL10), and C-X-C motif chemokine ligand 11 (CXCL11), which induce chemotaxis of other leukocytes, mainly activated T lymphocytes. 11 This cycle of recruitment of infected leukocytes, just as the high concentration of proinflammatory cytokines (known as “cytokine storm”), triggers a neuroinflammation condition. 11

### Direct nerve-ending infection

On the other hand, direct infection occurs when the virus invades the nerve endings and consequently reaches the CNS. Therefore, the pathogen does not need to cross the BBB. Neural access occurs through the nasal region, where SARS-CoV-2 present in the nasal endothelium adheres to the sensory and olfactory nerves, or through the lower respiratory tract, where the virus ascends through the vagus nerve. 2 3 8 Upon infecting nerve endings, the pathogen diffuses along synapses through endocytosis/exocytosis and axonal transport. Thus, the nasal route is evidenced by the clinical manifestation of anosmia (loss of smell), a common symptom in COVID-19 patients (the prevalence varies between 34% and 68% in some studies).

### Cytokine storm and microglia activation

Coronavirus disease 2019 triggers a strong inflammatory response in the host, known as a “cytokine storm,” with high levels of TNF-α, interleukin-6 (IL-6) and interferon-gamma (IFN-γ). 10 12 These cytokines are polypeptides or glycoproteins that act as inflammatory mediators controlling the immune response; when they are released by leukocytes, they are called interleukins. Thus, proinflammatory cytokines are produced by activated macrophages and recruit leukocytes to the site of infection. In the CNS, IL-6 is produced by astrocytes (cells responsible for supporting and nourishing neurons) and by microglia (cells of the neural defense system). It is worth mentioning that in some in vitro studies, 13 IL-6 destabilized the proteins that make up the juxtaposition of endothelial cells in the BBB, making it more permeable through the paracellular mechanism. So, the cytokine storm alters the optimal functioning of the BBB.

By invading the CNS, regardless of the access route, SARS-CoV-2 causes microglia activation. It is important to mention that activated glial cells are markers of neuropathologies, brain injuries, and neuroinflammation. 14 Although microglial cells are not the only cell type responsible for triggering brain inflammatory responses (systemic immune cells can also trigger neuroinflammation due to the release of proinflammatory cytokines into the bloodstream that cross the BBB), they respond quickly to environmental changes. 8 When these cells are activated, in addition to phagocytosing damaged cells, they secrete quinolinic acid, interleukins, complement system proteins, and TNF-α. In this context, increased quinolinic acid, an agonist of the N-methyl D-aspartate (NMDA) receptor, leads to neurotoxicity and can affect memory, learning, neuroplasticity, and cause hallucinations. 14 15

**Figure 2:**
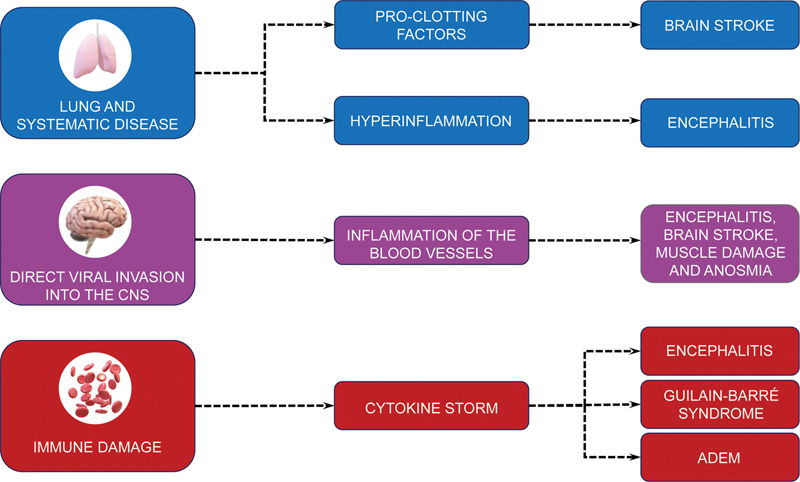
Abbreviation: ADEM, acute disseminated encephalomyelitis. Mechanism and neurological manifestations of COVID-19.

**Figure 3:**
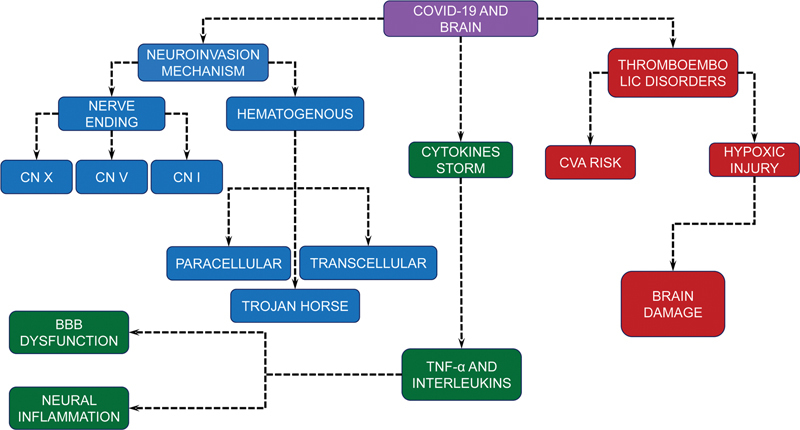
Abbreviations: CVA, cerebrovascular accident; CN, cranial nerve; BBB, blood-brain barrier. Mind map of the pathophysiological mechanisms by which COVID-19 affects the neurological system of the infected individual.

In conclusion, the neuroinflammatory state confers neuroprotection in the short term; however, it promotes neurodegeneration in a second moment ( Figure 2 , Figure 3 ). This will depend on the interactions between the release of pro- and antiinflammatory cytokines resulting from the viral infection. Thus, an exacerbated response of the host's immune system is capable of causing irreversible brain damage. 8 10

### Increased thrombotic factors

Patients with COVID-19 have high levels of procoagulation factors such as fibrinogen and prothrombin. 2 16 These disorders increase the risk of blockage of cerebral vessels, contributing to episodes of stroke ( Figure 3 ). From this perspective, autopsy studies 14 have shown that brain damage resulting from COVID-19 involves macro to micro hypoxic-ischemic lesions. This ischemic injury causes the death of neural cells, which can lead to multiple irreparable clinical outcomes, depending on the brain region affected. Thus, the presence of preexisting comorbidities (such as advanced age and cardiovascular diseases) is a significant risk factor for the occurrence of stroke in COVID-19 patients.

## BRAIN STUDIES IN AUTOPSIES OF COVID-19 PATIENTS

The study of autopsies in the brains of COVID-19 patients helps to describe how SARS-CoV-2 can directly or indirectly affect the CNS. Among the main macroscopic findings, it is possible to observe the presence of hemorrhagic lesions, atrophy, hydrocephalus, low brain weight, encephalitis, and stroke, while the main microscopic findings include hypoxic-ischemic damage, axonal swelling, myelin loss, gliosis, and microglial nodules. 2 17

## NEUROLOGICAL CLINICAL SYNDROMES OF COVID-19

Though COVID-19 mainly affects the respiratory tract, as aforementioned, the class of coronaviruses, including SARS-CoV-2, have neuroinvasive potential and neurotropic behavior ( Figure 2 , Figure 3 ). The most common neurological clinical syndromes include cerebrovascular events, encephalopathy, and cranial and peripheral neuropathies ( Figure 2 ). 9 18

### Cerebrovascular events

Cerebrovascular accident (CVA) occurs when there is an obstruction (ischemic condition) of the cerebral blood supply or when there is a rupture (hemorrhagic condition) of a cerebral vessel, causing the death of neuronal cells due to the interruption of the oxygen supply. As aforementioned, COVID-19 causes a state of hypercoagulability, evidenced by the high level of prothrombotic factors and fibrin degradation products in critically-ill patients, such as fibrinogen and D-dimer. 11 16 18 In this case, the risk of obstruction of blood vessels is considerably greater, which can lead to thrombosis of the arteries responsible for the cerebral blood supply. In many cases, the occlusion is not complete, but the sheer decrease in blood flow to the brain can cause irreversible damage to the nervous tissue due to hypoxia. In severe cases, complete obstruction may occur, leading to malignancy and possibly death. 11 18 19 20 Furthermore, the hypercoagulable state can also trigger cerebral venous thrombosis. 18 19 However, cases of this type are very rare, although there is a greater relative risk in COVID-19 patients. Recent retrospective studies 20 have demonstrated a significant prevalence (of around 3% to 6%) of cerebrovascular alterations in critically-ill patients, and more than a third of these patients died.

### Encephalopathies

Encephalopathy is any pathological process that affects the brain. Encephalitis is an encephalopathy characterized by brain inflammation related to any underlying insult. 19 This condition can be classified in three ways: infectious encephalitis, which is caused by the direct invasion of the brain by a microorganism; postinfectious encephalitis, which is caused by the host's immune response triggering an infection; and autoimmune encephalitis, which is not directly related to the infection. As aforementioned, COVID-19 can affect the brain directly through the virus or the immune response. Recent studies 18 have documented the incidence of infectious and postinfectious encephalitis in patients with SARS-CoV-2, a fact that is probably related to the alteration of the optimal functioning of the BBB as well as the cytokine storm. When brain inflammation (encephalitis) is not proven, other causes of the presence of encephalopathies should be considered, such as toxins, drugs, and hypoxemia (hypoxic encephalopathy). 9 The clinical manifestations of encephalopathies range from dizziness, mental confusion, seizures, and focal neurological deficits, to sensorium fluctuation or even coma. 20

### Acute disseminated encephalomyelitis and myelitis

Acute disseminated encephalomyelitis (ADEM) is a rare disease characterized by multifocal demyelination, usually manifesting after a viral infection. Myelitis is inflammation of the spinal cord due to (viral or bacterial) infections or autoimmune manifestations. In this perspective, case reports 21 22 have already described COVID-19 patients with ADEM or acute myelitis. However, the incidence rates of this group of neurological complications are not known for sure.

### Cranial nerve neuropathy

The involvement of the cranial nerves can manifest as cranial mononeuropathy or polyneuropathy, unilateral or bilateral, with or without the involvement of the peripheral nervous system (PNS) or the CNS. 23 Olfactory dysfunction is a frequent early symptom of COVID-19, reported in up to 80% of the patients in the first 5 days of illness. 24 25 26 Inferior cranial nerves may be additional sites of entry for the virus, causing early involvement of the lower brainstem, as well as the anatomical location of the respiratory center, which might explain some characteristics of COVID-19, such as hypoxia disproportionate to dyspnea and recurrent cases of syncope. 26 27 Neurological manifestations that occur concurrently with symptoms of COVID-19 suggest a possible mechanism of direct neural injury (neuroinvasion); neurological manifestations that follow symptoms of COVID-19 suggest indirect mechanisms, probably caused by the immune system. 26 27 28 Studies have analyzed the cerebrospinal fluid (CSF) for the investigation of SARS-CoV-2 RNA in patients with CNS/PNS involvement. Most found negative results, suggesting that overactivation of the immune system is the main pathophysiological mechanism. 29 Anosmia/hyposmia and ageusia/hypogeusia are attributable to the relationship of cranial nerves I, VII and IX. Auditory complications, both unilateral and bilateral, were described in some studies, as well as the isolated involvement of the XII cranial pair. 30 31

### Guillain-Barré syndrome

Guillain-Barre syndrome (GBS) is an autoimmune pathology that affects the nerve endings, characterized by an acute inflammatory polyradiculoneuropathy, and its most common clinical manifestations are paresthesia (tingling sensation) in the upper and lower limbs, flaccid muscle weakness ascending, dysphagia (difficulty on swallowing), and cranial nerve palsy. 19 32 Few case reports 19 20 33 have described the development of GBS in COVID-19 patients, which is an uncommon neurological manifestation of this disease.

### Nerve compression syndrome and critical illness polyneuropathy

Nerve compression syndrome is a pathological condition in which the peripheral nerves are compressed due to the patient's prolonged time in intensive care units (ICUs). Critical illness polyneuropathy is an axonal sensorimotor disorder that affects critically-ill patients admitted to the ICU. It is characterized by symmetrical distal skeletal muscle weakness, difficulty in weaning from ventilation with extubation failure, and generally length-dependent loss of sensation. The main cause of polyneuropathy is a systemic inflammatory syndrome, common in COVID-19 due to the cytokine storm. Studies 34 35 have reported the development of these complications in patients who remained hospitalized for long periods in the ICU, especially those who had received high doses of corticosteroids or had remained on neuromuscular blockers for several days.

## NEUROLOGICAL SYMPTOMS OF COVID-19

The key neurological symptoms related to COVID-19 include anosmia/hyposmia, ageusia/hypogeusia, dizziness, and headache. Table 1 points to studies involving numerous patients affected by the infection and the respective incidence of symptoms.

**Table 1:** Studies describing neurological manifestations in COVID-19 patients

Reference	Type of study	Population	Symptoms
Chen et al. (2020) 36	Retrospective review	99	Confusion (9%) and headache (8%)
Chen et al. (2020) 37	Retrospective study	113	Headache (11%) and dizziness (8%)
Giacomelli et al. (2020) 38	Cross-sectional study	59	Olfactory/or taste dysfunction (34%)
Hopkins et al. (2020) 39	Observational cohort	382	Anosmia (86%)
Huang et al. (2020) 40	Prospective study	41	Headache (8%)
Klok et al. (2020) 41	Retrospective review	184	CVA (1.6%)
Li et al. (2020) 42	Retrospective review	221	CVA (6%) and CVT (0.5%)
Lodigiani et al. (2020) 43	Retrospective review	338	CVA (2.5%)
Mao et al. (2020) 15	Retrospective review	214	Headache (13%), dizziness (17%), impaired consciousness (8%), CVA (3%), dysosmia (5%), and dysgeusia (6%)
Wan et al. (2020) 44	Retrospective review	135	Headache (35%)
Wang et al. (2020) 45	Retrospective review	138	Headache (7%) and dizziness (9%)
Wang et al. (2020) 46	Retrospective review	69	Headache (14%) and dizziness (7%)
Yang et al. (2020) 47	Retrospective review	52	Headache (6%)
D et al. (2021) 33	Meta-analysis	6,804	Guillain-Barré syndrome (0.8%)
D et al. (2021) 33	Meta-analysis	53,981	Encephalitis (0.1%)
D et al. (2021) 33	Meta-analysis	6,607	Encephalopathy (10.4%)
Finsterer et al. (2021) 34	Literature review	261	Nerve compression syndrome (7%)
Rifino et al. (2021) 48	Retrospective study	1,760	CVD (38%), CVT (0.7%), GBS (12.4%), CIP (6.6%), and encephalitis (3.6%)
Zhao et al. (2020) 22	Case report	1	Acute myelitis
Zhang et al. (2021) 21	Case report	1	ADEM

Abbreviations: ADEM, acute disseminated encephalomyelitis; CIP, critical illness polyneuropathy; CVA, cerebrovascular accident; CVD, cerebrovascular diseases; CVT, cerebral venous thrombosis; GBS, Guillain-Barré syndrome.

### Anosmia and hyposmia

The loss of olfactory capacity (anosmia) or simply its decrease (hyposmia) is deeply related to the SARS-CoV-2 neuroinvasion route through the nerve endings of the olfactory nerve in the nasal region. 2 10

### Ageusia and hypoageusia

As for the loss of the ability to taste (ageusia) or simply a decrease in it (hypogeusia), like the pathophysiological mechanism responsible for anosmia/hyposmia, it is believed that the olfactory, trigeminal and gustatory terminals are the main gateway for the virus to enter the CNS through retrograde axonal transport ( Figure 1 ). Alternative mechanisms of neuroinvasion, such as the hematogenous route, may also be responsible for these neurological manifestations.

### Headache

Headache is often described as an unspecific symptom, mainly in viral conditions. In patients infected with SARS-CoV-2, headache is a frequent complaint ( Table 1 ). 44 45 46 47 49 Recent publications 50 51 52 suggest a relationship between COVID-19 and intracranial hypertension, which, due to a continuous low-grade inflammation (cytokine storm), the increase in blood viscosity, and the state of hypercoagulability, can cause an increase in intracranial pressure. In a cross-sectional study, 52 56 patients underwent lumbar puncture for CSF analysis, and 13 of them manifested recurrent and persistent headaches, described as intense throbbing, holocranial or bilateral. About 84% (11/13) had intracranial hypertension in the absence of meningitis/encephalitis. 52

## NEUROPSYCHIATRIC MANIFESTATIONS OF COVID-19

As aforementioned, psychiatric manifestations can also be directly caused by the virus or by the host's immune response. However, these manifestations can also come from the psychological stress that the pandemic has caused in people, increasing anxiety disorders, depression, and poor sleep quality. Previous preclinical studies 2 14 16 have already documented the possible relationship between the proinflammatory immune response and the pathophysiology of diseases such as depression, anxiety, and bipolar disorder, relating high levels of TNF-α and certain cytokines to these conditions. In addition, a significant number of patients continue to experience these symptoms after the acute phase of the disease. This condition is described as a postCOVID syndrome, in which neuropsychiatric manifestations persist because of their involvement in psychological factors and neural damage. 53 54

Thus, although COVID-19 mainly affects the respiratory system, the neurotropism of SARS-CoV-2, its etiological agent, and its neuroinvasion mechanisms show its ability to affect the CNS. In general, there are fewer cases of patients with neurological manifestations when compared with respiratory manifestations. However, the hyperinflammatory response environment, resulting from the “cytokine storm” phenomenon, as well as thromboembolic events, represents a substantial risk to the nervous system. Therefore, physicians must pay attention to the neurological risks COVID-19 can pose for their patients.
